# Evidence of Human Exposure to Tamdy Virus, Northwest China

**DOI:** 10.3201/eid2712.203532

**Published:** 2021-12

**Authors:** Abulimiti Moming, Shu Shen, Yaohui Fang, Jingyuan Zhang, Yanfang Zhang, Shuang Tang, Tianxian Li, Zhihong Hu, Hualin Wang, Yujiang Zhang, Surong Sun, Lin-Fa Wang, Fei Deng

**Affiliations:** Wuhan Institute of Virology, Chinese Academy of Sciences, Wuhan, China (A. Moming, S. Shen, Y. Fang, J. Zhang, Y. Zhang, S. Tang, T. Li, Z. Hu, H. Wang, F. Deng);; College of Life Science and Technology, Xinjiang University, Urumqi, China (A. Moming, S. Sun);; National Virus Resource Center, Wuhan (S. Shen, Y. Zhang, S. Tang, F. Deng);; Center for Disease Control and Prevention of Xinjiang Uygur Autonomous Region, Urumqi, China (Y. Zhang);; Programme in Emerging Infectious Diseases, Duke-NUS Medical School, Singapore (L.F. Wang)

**Keywords:** Tamdy virus, arboviruses, human virus exposure, China, Orthonairovirus, tickborne virus, ticks, virus isolation, viruses

## Abstract

We report the isolation of Tamdy virus from *Hyalomma asiaticum* ticks in northwest China and serologic evidence of human Tamdy virus infection in the same region. These findings highlight the need to further investigate a potential causal relationship between Tamdy virus and febrile illnesses of unknown etiology in that region.

The species *Tamdy orthonairovirus* (genus *Orthonairovirus*, family *Nairoviridae*) includes 5 viruses: Tamdy virus (TAMV), Burana virus (BURV), Tǎchéng tick virus 1 (TcTV-1), Huángpí tick virus 1 (HpTV-1), and Wēnzhōu tick virus (WzTV) (*1*). TAMV and BURV were initially isolated from ticks in countries in central Asia (*2*–*4*), but little is known about their medical and veterinary importance. TcTV-1, HpTV-1, and WzTV were putative viruses identified by virome sequencing from ticks in China (*5*); however, their virologic properties and pathogenesis potential remain unclear. One study (*6*) reported TcTV-1 isolated from a febrile patient in northwest China, providing evidence of the potential public health threat from these viruses. We report TAMV isolated from ticks in northwest China and demonstrate serologic evidence of infection in humans. 

## The Study

**Figure 1:**
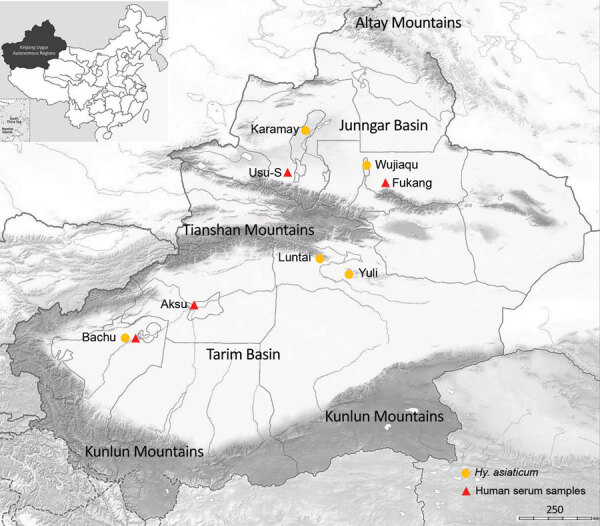
Collection locations for *Hyalomma asiaticum* ticks and human serum samples used in study of human exposure to Tamdy virus, Xinjiang, China. Usu-S, southern area of Usu City.

During April and May of 2016 and 2017, we collected *Hyalomma asiaticum* ticks (n = 4,123) from Xinjiang in northwest China and divided the ticks into 55 groups according to the sampling location (n = 50–100 ticks/group) (Figure 1; Appendix Table 1). We isolated the virus from homogenates of each tick group in suckling mice. After the first inoculation, we observed symptoms in mice including loss of balance, limb paralysis, tremors, and articulo mortis from 4 (36.37%) of 11 pooled samples from Yuli County, 1 (14.29%) of 7 from the city of Karamay, 3 (60%) of 5 from Luntai County, and 17 (53.13%) of 32 from the city of Wujiaqu (Appendix Table 1). We performed a second inoculation using brain samples from diseased mice from Luntai and Wujiaqu Counties, in which >50% of the mice experienced illness onset after first inoculation. Similar symptoms were reproducibly observed in 1 group from Luntai and 4 groups from Wujiaqu (Appendix Table 1). 

Subsequently, we prepared 3 RNA pools of diseased mouse brains and obtained a total of 196,946,814 reads by RNA sequencing. We found TAMV contigs in all 3 pools (Appendix Table 2), confirming findings using real-time reverse transcription PCR (rRT-PCR) (data not shown). We used homogenates of 2 TAMV RNA-positive brain samples from the A-M6 pool (Appendix Table 2) to isolate viruses in Vero E6 cells. As indicated by immunofluorescence assays (IFA) (Appendix), we observed increasing TAMV infection from first to fourth passages in Vero E6 cells, suggesting successful isolation (Appendix Figure 1).

**Figure 2:**
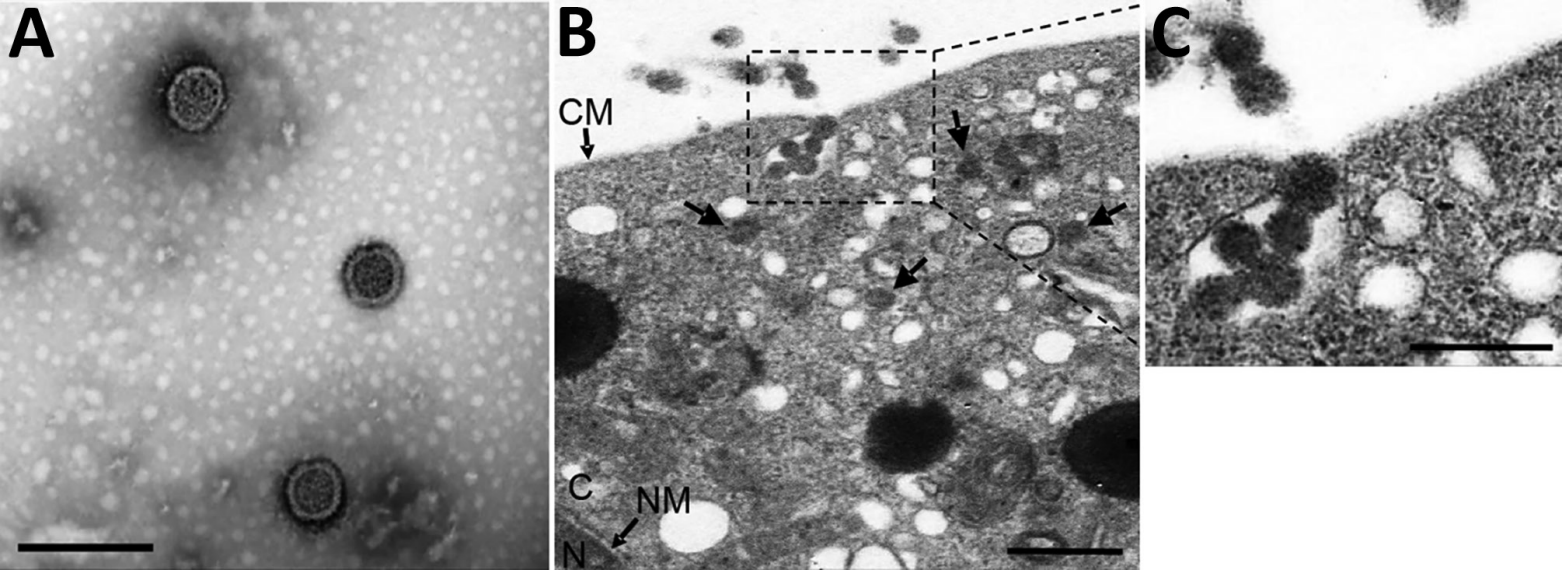
Visualization and subcellular localization of Tamdy virus (TAMV) virions by electron microscopy. A) Negative-staining image of purified TAMV virions. Scale bar indicates 200 nm. B) Image of Vero E6 cells infected with TAMV; arrows indicate TAMV virions in the cytoplasm. Scale bar indicates 500 nm. C) The enlarged image of interest from B. scale bar indicates 200 nm. CM, cell membrane; C, cytoplasm; NM, nuclear membrane; N, nucleus.

Negative-stain electron microscopy revealed an enveloped spherical viral morphology with a diameter of ≈90–110 nm (Figure 2, panel A). We observed viral particles in cytoplasm and vesicles of infected cells (Figure 2, panel B). Although this screening was not exhaustive, IFAs showing varied susceptibility of different cells lines indicate that TAMV seems to have a broad host range, including humans, monkeys, sheep, dogs, and mice (Appendix Figure 2).

The 2 TAMV isolates shared very high sequence similarities (99.96% for large, 100% for medium, and 99.95% for small segments). We named the 2 TAMV strains YL16082 and YL16083, including an abbreviation (YL) for the geographic location (Yuli County) where the original tick samples were collected. TAMV genome sequences (YL16082) shared 37%–59% identity with other members of the *T. orthonairovirus* species and 34%–49% identity with Crimean-Congo hemorrhagic fever virus (CCHFV), another *Orthonairovirus* species. Protein sequences shared 44%–62% identity with other members of the *T. orthonairovirus* species and 33%–40% with CCHFV (Table 1). Phylogenetic trees based on nucleotide sequences of RNA-dependent RNA polymerase, glycoprotein, and nucleoprotein (NP) genes all confirmed the close taxonomic relationships with currently known TAMV strains and other members of the species *T. orthonairovirus* (Appendix Figure 3).

**Table 1:** Sequence identity of TAMV isolate YL16082 from China compared with other members of the species *Tamdy orthonairovirus* and Crimean-Congo hemorrhagic fever virus*

Virus	Nucleotide identity, %				Amino acid identity, %		
	L segment	M segment	S segment		RdRp	G	NP
Wēnzhōu tick virus	59	50	42		62	51	44
Tǎchéng tick virus 1	57	46	44		60	51	49
Huángpí tick virus 1	55	45	42		58	47	46
Burana virus†	59	47	37		62	50	44
Crimean-Congo hemorrhagic fever virus	49	34	39		40	33	36

*G, glycoprotein; L, large; M, medium; NP, nucleoprotein; RdRp, RNA-dependent RNA polymerase; S, small; TAMV, Tamdy virus. †Partial sequences were available for analyses.

To investigate potential human infection by TAMV in northwest China, we conducted a seroprevalence study using archived serum samples from 725 healthy persons (collected in 2005 from Fukang City, 2014 from Aksu City, and 2017 from Usu City) and 87 febrile patients (collected in 2007 from Bachu County, which has a history of CCHFV prevalence) (Appendix). Of the 87 febrile patients, 21 (24.14%) were TAMV IgG positive and 17 (19.54%) IgM positive (Table 2; Appendix Table 4), whereas only 1 (0.13%) of the 725 healthy participants we tested IgG positive (data not shown). Neutralization (titers 16–64) was demonstrated in serum samples from 6 febrile patients (6.9%) (Table 2). Moreover, of the 24 tick groups from the same locations as the febrile patients, 10 groups (41.76%, 6 identified in sheep and 4 in fields) tested positive for TAMV RNA by rRT-PCR (data not shown). Partial sequences of large segments from these positive groups clustered together with TAMV strains (Appendix Figure 4). These results showed serologic evidence of human exposure to TAMV and evidence of TAMV presence in *Hy. asiaticum* ticks in northwest China as early as 2007, which warranted more in-depth investigation to establish the potential causal relationship between TAMV and febrile illnesses of unknown etiology in regions where TAMV is present.

**Table 2:** Seroprevalence of TAMV among 87 febrile patients with samples collected in 2007 from Bachu County, Xinjiang, China*

Patient characteristics	Serum samples, no.	TAMV IgG positive, %	TAMV IgM positive, %	Neutralization activity, %	Neutralization titers
Sex					
M	20	3 (15.0)	7 (35.0)	1 (5.0)	64
F	29	6 (20.7)	5 (17.2)	3 (10.3)	16, 32, 32
NA	38	12 (31.6)	5 (13.6)	2 (5.3)	16, 16
Age, y†					
<18	6	2 (33.3)	1 (16.7)	0	
18–30	20	1 (5.0)	8 (40.0)	1 (5.0)	16
31–45	7	2 (28.6)	1 (14.3)	0	
46–60	11	2 (18.2)	0	1 (9.1)	32
>60	5	2 (40.0)	2 (40.0)	2 (40.0)	32, 64
Total	87	21 (24.1)	17 (19.5)	6 (6.9)	16–64

*NA, not available; TAMV, Tamdy virus. †Age stratification based on 49 patients with recorded age information

Finally, because another *T. orthonairovirus*, TcTV-1, had been identified in a febrile patient in northwest China (*6*), we thought it important to determine the potential serologic cross-reactivity between these 2 viruses. However, TAMV and TcTV-1 shared limited protein sequence identity (49%–60%) (Table 1), suggesting limited cross-reactivity, if any. This result was confirmed by conducting serologic testing using recombinant NP proteins from the 2 viruses. As shown by both IFA and Western blot analyses, TAMV NP antibodies had no cross-reaction with TcTV-1 NP (Appendix Figure 5, panels A, B). In addition, human serum samples that were positive for TAMV IgM or IgG, or both, showed reactivity with TAMV NP, but not with TcTV-1 NP (Appendix Figure 5, panel C).

## Conclusions

TAMV was initially found in ticks in countries in central Asia, including Kazakhstan, Kyrgyzstan, Uzbekistan, and Turkmenistan (*3*,*4*). Its infection status in humans and livestock animals was not well characterized. Our data, together with reports of TAMV isolated from ticks in Xinjiang, China (*7*), and identified in Turkey (*8*), shows that the geographic distribution of TAMV is much wider than originally recognized. In addition, we provide strong serologic evidence of human exposure in TAMV-affected regions.

Findings of a potential role of TcTV-1 in causing human febrile disease (*6*) suggest that >1 virus in this species group may have the potential to cause diseases in humans. However, our data did suggest the possibility of such a relationship because the TAMV-positive ratio was much higher among febrile patients than healthy persons in the study from the same region. In addition, at least 2 febrile patients had both TAMV IgM and IgG at the time of sampling, during or not long after acute illness; 1 of them had neutralization to TAMV (Appendix Table 3).

Among study limitations, the nature of using archived samples limited our ability to provide direct evidence of a causal relationship between TAMV and human febrile illnesses. Also, it is possible that the high TAMV antibody-positive ratio might have resulted not from the recent cases but from a small outbreak of human TAMV infection in northwest China in 2007.

In summary, our study strongly suggests the potential of TAMV as a human pathogen and supports an urgent need to conduct more in-depth epidemiologic and pathogenesis investigations into this group of viruses in China, central Asia, and beyond. While the world’s attention is currently on coronavirus disease and batborne viruses, our study highlights the need to pay attention at the same time to emerging zoonoses of tick origin to prevent future outbreaks.

Appendix Additional information on human exposure to Tamdy virus in China.
