# Supplementary material for: Evidence of Human Exposure to Tamdy Virus, Northwest China
Source: Emerg Infect Dis. 2021 Dec;27(12):3166–70. doi: 10.3201/eid2712.203532 (PMC8632163; doi:10.3201/eid2712.203532)
Supplement: Appendix — Additional information on human exposure to Tamdy virus in China. [file 20-3532-Techapp-s1.pdf]

Article DOI: https://doi.org/10.3201/eid2712.203532

Evidence of Human Exposure to Tamdy Virus, Northwest China

Appendix

Collection of ticks in Xinjiang, China

In April and May of 2016 and 2017, we collected a total of 4,123 *Hyalomma asiaticum* ticks from wild fields locating in Karamay City, Yuli County, Luntai County, and Wujiaqu City in Xinjiang using methods described elsewhere (*1*) (Figure 1). We first identified tick species by morphologic characteristics under microscope and confirmed identifications using sequencing of the rRNA internal transcribed spacer 2 gene, as described elsewhere (*2*). We divided the ticks into 55 groups (n = 50–100 ticks/group) by sampling locations. We prepared homogenates of the 55 tick groups and used them to inoculate mice as described elsewhere (*3*) (Appendix Table 1). In addition, 24 groups of *Hy. asiaticum* ticks (17 groups each from different individual sheep and 7 from wild fields) were collected in 2007 from Bachu, the same origin as archived samples from the febrile patients were collected (Figure 1).

Virus Isolation by Mouse Inoculation and Molecular Characterization

We isolated viruses by mouse inoculation as described elsewhere (*3*) in an animal biosafety level 2 laboratory according to the animal welfare and related regulations specified in the Directory of Pathogenic Microorganisms Transmitted among Humans issued by the Chinese Ministry of Health (https://www.nhc.gov.cn/wjw/gfxwj/201304/64601962954745c1929e814462d0746c.shtml). We harvested the brains of diseased mice and stored them in glycerol at –80°C.

We identified the viruses in the mouse brains using RNA-seq. We generated clarified homogenates from the brain of 1 diseased mouse randomly selected from each group as described elsewhere (*3*). We purified total RNA from 250 μL of clarified brain homogenates using TRIzol (Invitrogen, https://www.thermofisher.com) according to manufacture instructions.

We prepared 3 RNA pools, each containing a mixture of 5 μg total RNA from the mouse brain samples (Appendix Table 1). We reverse transcribed the rest of the RNA from each mouse brain into complementary DNA (cDNA) and stored it at –80°C until used for further investigation. RNA-seq library preparation was performed according to the protocol provided by Illumina (*4*). We used HiSeq 3000 platform (Illumina, https://www.illumina.com) for 150 bp paired-end sequencing of the libraries, and analyzed sequencing data to identify viral-related sequences as described elsewhere (*5*).

For rRT-PCR analysis of TAMV-specific viral RNA, we purified total RNA from homogenates from mouse brains (100 μL), tick groups (100 μL), or cell culture supernatants (300 μL) using TRIzol (Invitrogen), and further transcribed into cDNA using M-MLV reverse transcription (TaKaRa Biotechnology, https://www.takarabio.com) according to manufacturer instructions. We performed PCR with primers TAMV-L-F: 5′-CTCAATCATCATCCTCGTCTCAT-3′ and TAMV-L-R: 5′-GGACAATAGTTCGCTTAGGCTCT-3′ to amplify a partial (423 nt) fragment of the L segment in a 50 μL reaction volume containing 1–3 μL cDNA templates.

Characterization of TAMV in Cell Culture Using Electron Microscopy

We isolated viruses by cell culture as described elsewhere (*2*) in a biosafety level 3 laboratory according to the Directory of Pathogenic Microorganisms Transmitted among Humans. We inoculated Vero E6 cells with 200 μL of clarified brain homogenates for 2 h at 37°C. We removed supernatants and replaced them with fresh GIBCO Dulbecco's Modified Eagle Medium (ThermoFisher) containing GIBCO 2% fetal calf serum (ThermoFisher) and continued incubation for 5–7 days at 37°C. We monitored infection susceptibility and virus replication using immunofluorescence assays (IFA) as described elsewhere (*2*) using the polyclonal α-TAMV-NP serum produced in-house (see below).

To test susceptibility of cell lines derived from different hosts to TAMV, U87MG, SW13, 293, Vero E6, MODK, MDBK, PK15, DH82, and BHK21 cells were incubated with 500 μL of clarified supernatants from fourth passage (P4) in Vero E6 cells. Virus infection was confirmed by IFA. For morphologic studies, we purified TAMV from supernatants and visualized it using

negative-staining electron microscopy as described (*1*). We examined subcellular localization of viral particles in Vero E6 cells by transmission electron microscopy as described elsewhere (*1*).

Cells, Viruses, and Antibodies

We purchased cell lines derived from human (U87MG [HTB-14], SW13 [CCL-105], and 293 [CRL-1573]), monkey (Vero E6 [CRL-1586]), sheep (MDOK [CRL-1633]), cattle (MDBK [CCL-22]), swine (PK15 [CCL-33]), dog (DH82 [CRL-10389]), and mouse (BHK-21 [CCL-10]) cells from American Type Culture Collection. The 2 isolates of TAMV are deposited in the National Virus Resource Center (IVCAS 6.7499 for strain YL16082; IVCAS 6.7500 for strain YL16083). We performed IFAs, western blots, and neutralization assays using the YL16082 isolate. We used mouse anti-hemagglutinin tag polyclonal antibody (α-HA) for IFAs as the primary antibody (ABclonal Technology, https://abclonal.com). We used β-actin antibody (α-β-actin) (Sangon Biotech, http://www.life-biotech.com) as the control for western blots. We used fluorescein isothiocyanate–conjugated sheep anti-human IgG antibody, rabbit anti-human IgM μ chain (Alexa Fluor 488, ThermoFisher), goat anti-mouse IgG H&L (Alexa Fluor 488, ThermoFisher), and goat anti-mouse IgG H&L (horseradish peroxidase) as secondary antibodies for IFAs and western blots (Abcam, https://www.abcam.com).

In-house Production of α-TAMV-NP in Mice

We amplified the 1,452 bp open reading frame coding region of TAMV NP from strain YL16082 from TAMV-positive mouse brain by PCR using 2×Rapid Taq Master Mix (Vazyme Biotech, https://www.vazymebiotech.com) according to manufacture instructions. We cloned PCR products into the plasmid pET-32a to generate the expression plasmid pET-32a-TAMV-NP and confirmed the insert by sequencing. We conducted protein expression, purification, and rabbit immunization as described elsewhere (*1*).

Serologic Investigation of Human Infection by TAMV

Using serum samples archived in a repository, we investigated TAMV seroprevalence in Xinjiang among 725 healthy persons: 465 from a southern area of Usu City in 2017 (*1*), 80 from Fukang City in 2005, and 180 from Aksu City in 2014, and in June 2007, among 87 febrile

patients from 1 hospital in the downtown area and 2 clinics in the village with the largest population in Bachu County. The 87 serum samples tested negative for Crimean-Congo hemorrhagic fever virus RNA and antibodies (data not shown). We recorded sex and age except from 38 febrile patients whose records lacked personal information. We tested for IgG and IgM with IFA as described elsewhere (*1*) using TAMV infected cells as antigen and α-TAMV-NP as the positive control antibody. We performed microneutralization tests using the TAMV strain YL16082 as described elsewhere (*1*).

Cross-Reactivity between TAMV Antiserum and TcTV-1 Proteins.

The protein sequence comparison between the 2 viruses revealed a sequence identity of ≈48%–60% (Table 1). We cloned full-length open reading frames of both TAMV (1,452nt) and TcTV-1 NP (1,473 nt) proteins in pCAGGS-P7 vector, generating the TAMV-NP expression plasmid and the TcTV-1-NP expression plasmid fused with HA tag at C-terminus (TcTV-1-NP-HA). We transfected Vero E6 cells with TAMV-NP expression plasmid, TcTV-1-NP-HA expression plasmid, and the control vector pCAGGS-P7. At 48 h after transfection, we conducted IFA using plasmid-transfected cells as described elsewhere (*3*) with α-TAMV-NP, α-HA, and IFA-positive serum samples from febrile patients. For western blot analyses, we also harvested plasmid transfected cells and blotted them with α-TAMV-NP, α-HA, and α-β-actin.

Sequences and Bioinformatics

We deposited the complete genome sequences of 2 TAMV strains in GenBank under the accession numbers from MT815989 to MT815994. The datasets of sequence reads from 3 pools of mouse brains are publicly available in the NCBI Sequence Read Archive (http://www.ncbi.nlm.nih.gov) under the ID PRJNA649646.

We used the coding regions of the L, M, and S segments of viruses from the genus *Orthonairovirus*, family *Nairoviridae*, to construct the maximum likelihood trees. We used sequences of severe fever with thrombocytopenia virus, Rift Valley fever virus, and Hantaan virus as outgroup sequences. We built the maximum likelihood tree of TAMV strains using the 423 nt partial L sequences obtained from PCR products for TAMV-positive tick groups. We

constructed trees using Mega 6.0 (6) and tested them using the bootstrap method with 1,000 replications.

Ethical Statement

Animal inoculation experiments and human serologic investigation were approved by the ethics committee of Wuhan Institute of Virology, Chinese Academy of Sciences under the approval numbers WIVA33201702 and WIVH01201501.

References

<jrn>1. Shen S, Duan X, Wang B, Zhu L, Zhang Y, Zhang J, et al. A novel tick-borne phlebovirus, closely related to severe fever with thrombocytopenia syndrome virus and Heartland virus, is a potential pathogen. Emerg Microbes Infect. 2018;7:95. PubMed https://doi.org/10.1038/s41426-018-0093-2</jrn>

<jrn>2. Lu X, Lin XD, Wang JB, Qin XC, Tian JH, Guo WP, et al. Molecular survey of hard ticks in endemic areas of tick-borne diseases in China. Ticks Tick Borne Dis. 2013;4:288–96. PubMed https://doi.org/10.1016/j.ttbdis.2013.01.003</jrn>

<jrn>3. Zhang Y, Shen S, Fang Y, Liu J, Su Z, Liang J, et al. Isolation, characterization, and phylogenetic analysis of two new Crimean-Congo hemorrhagic fever virus strains from the northern region of Xinjiang Province, China. Virol Sin. 2018;33:74–86. PubMed https://doi.org/10.1007/s12250-018-0020-7</jrn>

<jrn>4. Li CX, Shi M, Tian JH, Lin XD, Kang YJ, Chen LJ, et al. Unprecedented genomic diversity of RNA viruses in arthropods reveals the ancestry of negative-sense RNA viruses. eLife. 2015;4:e05378. PubMed https://doi.org/10.7554/eLife.05378</jrn>

<jrn>5. Shi M, Lin XD, Tian JH, Chen LJ, Chen X, Li CX, et al. Redefining the invertebrate RNA virosphere. Nature. 2016;540:539–43. PubMed https://doi.org/10.1038/nature20167</jrn>

<jrn>6. Tamura K, Stecher G, Peterson D, Filipski A, Kumar S. MEGA6: Molecular evolutionary genetics analysis version 6.0. Mol Biol Evol. 2013;30:2725–9. PubMed https://doi.org/10.1093/molbev/mst197</jrn>

<jrn>7. Zhou H, Ma Z, Hu T, Bi Y, Mamuti A, Yu R, et al. Tamdy virus in *Ixodid* ticks infesting bactrian camels, Xinjiang, China, 2018. Emerg Infect Dis. 2019;25:2136–8. PubMed https://doi.org/10.3201/eid2511.190512</jrn>

Appendix Table 1. Summary of tick samples used for mouse inoculation*

Year	Location	Ticks	Tick groups, no.	Mouse groups showing illness at P1	Mouse groups for further inoculation	Mouse groups showing illness at P2
2016	Yuli County	550	11	4	NA	NA
	Karamay City	383	7	1	NA	NA
	Luntai County	250	5	3	1	1
2017	Wujiaqu City	2,940	32	17	6	4
Total		4,123	55	25	7	4

*NA, not applicable; P1, first inoculation; P2, second inoculation.

Appendix Table 2. Summary of RNA pools of mouse brains used for RNA sequencing

RNA pool	Mouse brains, no.	Location of ticks used to inoculate mice	Generation of inoculated mice	Total reads	TAMV-related reads	nt Sequence identity to TAMV, %
A-M6	4	Yuli County	P1	52,476,380	64,425	94.92–96.96
	1	Karamay City	P1			
ML	4	Luntai County	P1 (×3)<br>P2 (×1)	95,862,588	382,724	94.80–96.86
XJ4	4	Wujiaqu City	P2	48,607,846	69,965	94.75–96.55
Total				196,946,814	517,114	

Appendix Table 3. Personal and clinical characteristics of febrile patients from Bachu County, June 2007*

Case	Sex/age, y	Inpatient/ outpatient	Clinical diagnosis and parameters	IFA		Neutralization, titer
				TAMV IgM	TAMV IgG	
1	M/30	O	Liver damage	–	–	NA
2	M/45	I	Hyperlipemia	–	–	NA
3	F/50	I	Coronary heart disease; liver damage	–	–	NA
4	F/18	O	Liver damage	–	–	NA
5	M/6	I	Liver damage	+	–	NA
6	F/21	O	Leukopenia (leukocyte: 9.6×10^8/L); liver damage	+	–	NA
7	F/22	O	NA	+	–	16
8	NA	O	Postpartum infection	+	–	NA
9	F/16	O	NA	–	–	NA
10	NA	O	NA	+	+	NA
11	F18	O	NA	+	–	NA
12	NA	O	NA	–	+	NA
13	NA	O	NA	–	–	NA
14	F/24	O	NA	–	-	NA
15	M/21	O	NA	+	-	NA
16	F/23	O	NA	–	–	NA
17	F/22	O	NA	–	–	NA
18	M/23	O	NA	+	–	NA
19	M/27	O	NA	+	–	NA
20	M/28	O	NA	+	–	NA
21	M/28	O	NA	+	–	NA
22	NA	O	NA	+	–	NA
23	F/29	I	NA	–	–	NA
24	F/25	I	NA	–	–	NA
25	F/15	O	NA	–	+	NA
26	NA	O	NA	–	–	NA
27	NA	O	NA	–	–	NA
28	NA	O	NA	+	–	NA
29	NA	O	NA	–	–	NA
30	NA	O	NA	–	–	NA
31	NA	O	NA	–	–	NA
32	NA	O	NA	+	–	NA
33	NA	O	NA	–	–	NA
34	M/50	I	NA	–	–	NA
35	NA	O	NA	–	+	NA
36	NA	O	NA	–	+	NA
37	NA	O	NA	–	–	NA
38	NA	O	NA	–	–	NA
39	NA	O	NA	–	–	NA
40	NA	O	NA	–	–	NA
41	NA	O	NA	–	–	NA
42	NA	O	NA	–	–	NA
43	NA	O	NA	–	–	NA

Case	Sex/age, y	Inpatient/ outpatient	Clinical diagnosis and parameters	IFA		Neutralization, titer
				TAMV IgM	TAMV IgG	
44	NA	O	NA	–	–	NA
45	F/21	I	Urticaria	–	–	NA
46	M/60	I	Gastritis	–	–	NA
47	M/41	O	Hyperlipidemia, hyperglycemia, coronary heart disease, renal dysfunction	–	+	NA
48	NA	O	NA	–	+	NA
49	NA	O	NA	–	–	NA
50	F/1	O	Rickets	–	–	NA
51	F/20	O	Suspected hepatitis	–	–	NA
52	M/45	O	Hypertension	–	–	NA
53	M/39	O	Hyperlipidemia, hyperglycemia	–	–	NA
54	F/30	I	Suspected chickenpox	–	–	NA
55	M/50	O	NA	–	–	NA
56	F/35	O	NA	+	–	NA
57	M/71	O	NA	–	–	NA
58	F/75	O	Hypertension	+	–	32
59	F/55	I	NA	–	–	NA
60	NA	O	NA	–	+	NA
61	NA	O	NA	–	+	16
62	NA	O	NA	–	–	NA
63	M/49	I	burn	–	–	NA
64	M/53	O	NA	–	–	NA
65	F/20	O	NA	–	–	NA
66	F/60	O	NA	–	–	NA
67	NA	O	NA	–	–	NA
68	F/40	O	Cholecystitis	–	–	NA
69	NA	I	NA	–	+	NA
70	F/56	I	NA	–	+	32
71	M/15	I	NA	–	+	NA
72	M/70	I	NA	+	+	64
73	M/62	I	NA	–	–	NA
74	F/15	I	NA	–	–	NA
75	NA	I	NA	–	–	NA
76	F/61	I	NA	–	+	NA
77	F/51	I	NA	–	–	NA
78	F/42	I	NA	–	+	NA
79	F/20	I	NA	–	+	NA
80	NA	I	NA	–	+	NA
81	NA	O	NA	–	+	NA
82	NA	O	NA	–	+	16
83	F/55	I	NA	–	+	NA
84	NA	O	NA	–	+	NA
85	NA	O	NA	–	–	NA
86	NA	I	NA	–	–	NA
87	NA	I	NA	–	–	NA
Total	20 M, 29 F, 38NA/1–75	26 I, 61 O	NA	17 (19.5%)	21 (24.1%)	6 (6.9%)

*IFA, immunofluorescence assays; NA, not available; I, inpatient; O, outpatient; TAMV, Tamdy virus; +, positive for antibodies; –, negative for antibodies

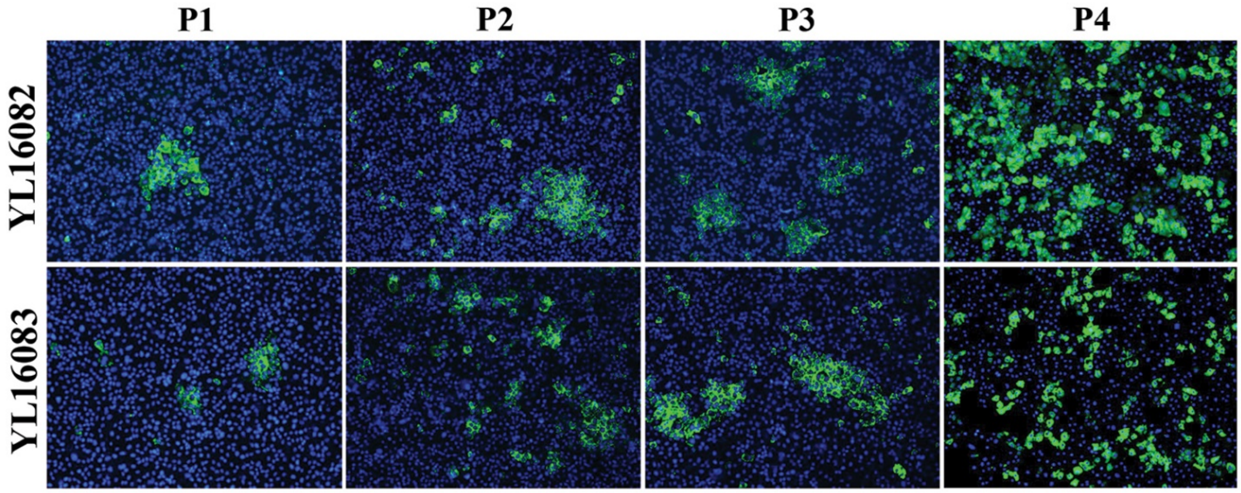

Appendix Figure 1. Isolation of 2 new TAMV strains (YL16082 and YL16083) by incubating clarified homogenates of suckling mouse brains in Vero E6 cells. We monitored productive virus replication using immunofluorescence assays for each passage (P1–P4) with a TAMV-NP antiserum.

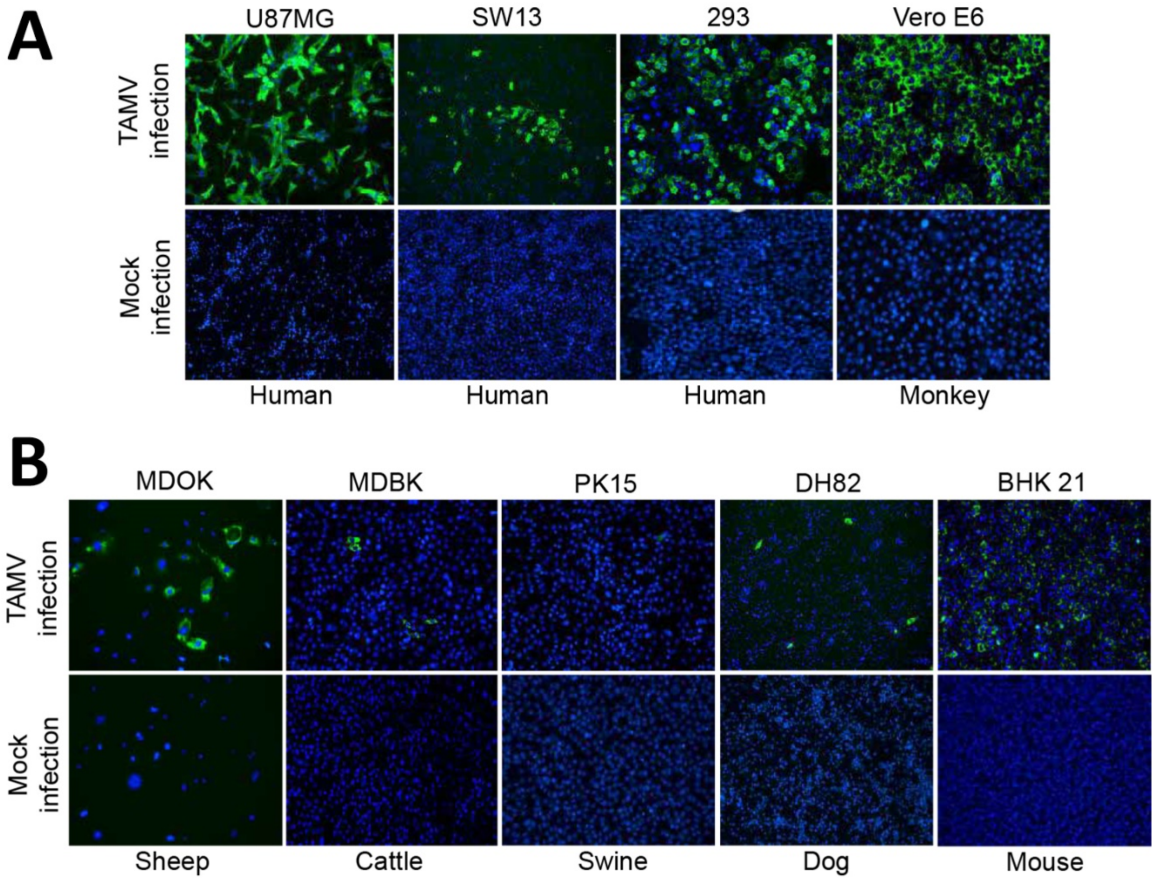

Appendix Figure 2. Infection susceptibility of different cells revealed by immunofluorescence staining. These include 3 different human cell lines and 1 each from monkeys, sheep, cattle, swine, dogs, and mice.

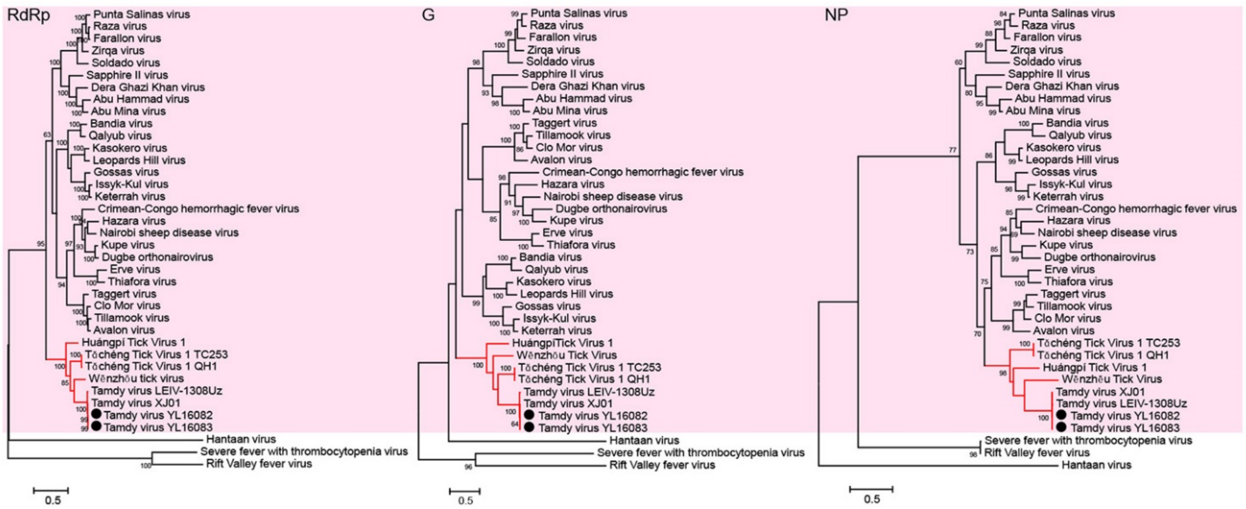

Appendix Table 3. Maximum likelihood phylogenetic trees based on the complete amino acid sequences of the RNA-dependent RNA polymerase (RdRp), glycoprotein (G), and nucleoprotein (NP) of virus members in the genus *Orthonairovirus* (shaded in pink), family *Nairoviridae*. The clade of *Tamdy orthonairovirus* species is shown by red lines. The 2 new TAMV isolates are labeled by black dots. Trees were constructed by bootstrap method of 1000 replicates.

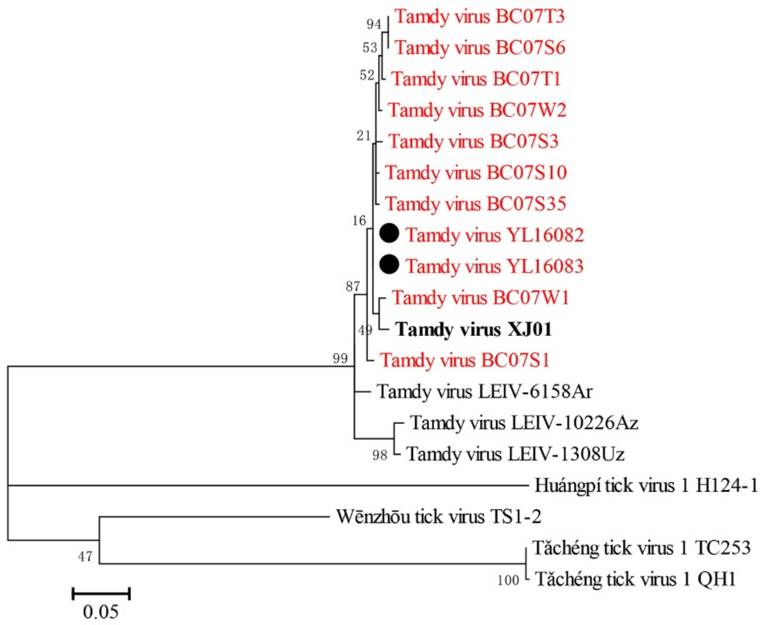

Appendix Figure 4. Maximum likelihood tree based on the partial (423 nt) sequences of the L segment of species *Tamdy orthonairovirus*. TAMV strains identified in this study are indicated by red characters; the 2 isolates (YL16082 and YL16083) are indicated by black dots. TAMV strain XJ01, identified in a recent report (*7*), is indicated in bold. Trees were constructed using a bootstrap method of 1000 replicates.

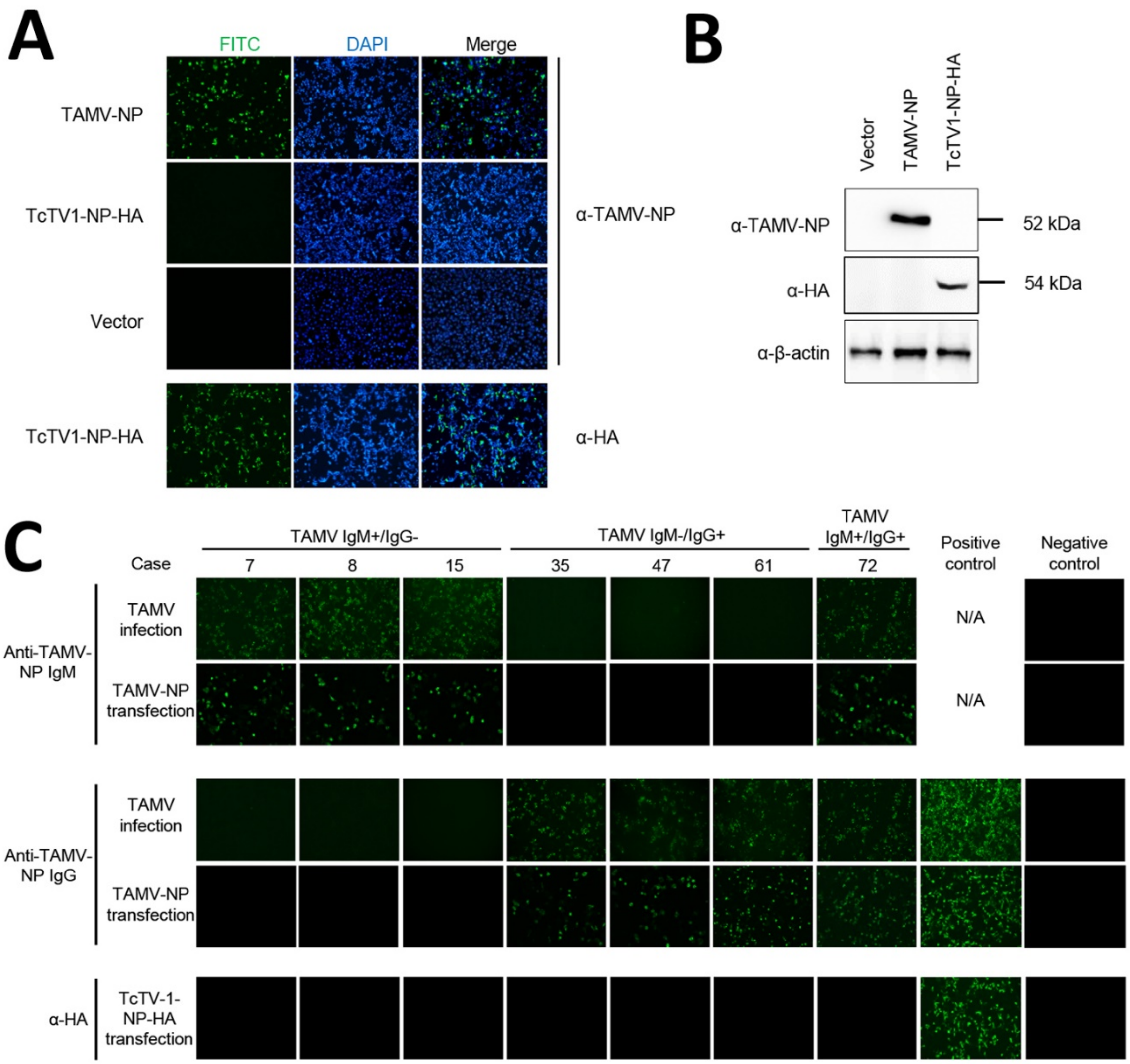

Appendix Figure 5. Examination of potential cross-reaction between TAMV NP polyclonal antibodies and Tăchéng tick virus 1 (TcTV-1) NP using A) immunofluorescence assays (IFA) and B) western blot. We transfected Vero E6 cells with TAMV-NP expression plasmid, TcTV-1-NP-HA expression plasmid, and control plasmid. At 48 hours after infection, we immune-stained fixed cells using α-TAMV-NP. We immune-stained cells transfected with TcTV-1 NP-HA expression plasmid using α-HA as a control. Transfected cells were harvested and detected by western blot using α-TAMV-NP, α-HA, and α-β-actin. C) Examination of reactivity of serum samples from febrile patients toward TAMV NP and TcTV-1 NP by IFA using virus-infected cells or plasmid-transfected cells. Representative images of serum samples from 7 febrile patients, with 3 IgM-positive samples (case-patients 7, 8, and 15), 3 IgG-positive samples (case-patients 35, 47, and 61), and 1 sample positive for both IgG and IgM (case-patient 72), determined using IFA with TAMV-infected cells. The upper panel shows images from the 7 samples being examined by α-TAMV-NP IgM using both TAMV-infected cells and TAMV NP transfected cells as antigens; the middle panel shows images from the examination of α-TAMV-NP IgG; and the bottom panel shows images from detection of TcTV-1 NP antibodies using TcTV-1-NP-HA transfected cells. NA, not applicable.
